# The Relationship among Community Environment, Behavior, Activity Ability, and Self-Rated Health of Older Adults: A Hierarchical and Multi-Dimensional Comparative Study

**DOI:** 10.3390/ijerph18147387

**Published:** 2021-07-10

**Authors:** Zhenhua Zheng, Hong Chen

**Affiliations:** 1 College of Communication and Art Design, University of Shanghai for Science and Technology, No. 516 Jungong Road, Shanghai 200093, China; zhenhuazheng@usst.edu.cn; 2 College of Architecture & Environment, Sichuan University, No. 24 First South Section First Ring Road, Chengdu 610065, China

**Keywords:** community environment, walking, neighbor contacts, self-rated health, activity ability, age group, older adults

## Abstract

Although the community environment is a known determinant of older adults’ health, it is unclear about the logical relationships among the community environment, behavior, activity ability, and health of older adults, and the differences between the different age groups. This study used a two-stage sampling method to conduct a household survey of people over 60 years old living in Xinhua Street, Shanghai, China. In total, 2783 valid samples were obtained. Of these, 1256 were males and 1627 were females, with an average age of 71.1 years. The statistical method used in this study was the structural equation modeling method. The effects of the community environment and behavior on the activity ability and self-rated health of older adults are different, and the path of health influence of older adults is different in different age groups. Community environment has more wider effects on older adults’ self-rated health, while behavior, including walking behavior and neighbor contacts, have a more intensive effect on the activity ability of older adults. The community environment has a significant positive effect on the activity ability of the younger group but not on that of the older group, which instead was significantly affected by the neighbor contacts. Therefore, refined environmental governance and targeted improvement and resolution of different types of health problems among different groups of older persons will contribute to the overall health of older adults.

## 1. Introduction

Population ageing is the current world problem. The U.N. (United Nations) predicted that by 2050, the number of people over 60 years old will reach 2.5 billion, accounting for about 21.3% of the population [1]. The health problems of the aged not only affect the resolve of the problems of support for the aged and the economic expenditure of different countries, but also determine the health level and the life quality of the population in every country and even the whole world.

The community is the main activity place and living space for older adults in the city. A good community environment contributes to the improvement of the health level of older adults [2,3,4]. Therefore, to improve the health of the aged, it is key to study how the community environment affects their health. In 2007, WHO (World Health Organization) put forward the concept of an “age-friendly community” and emphasized the promotion of greater social interaction, more meaningful activities, and better health opportunities for older persons through the improvement of the physical environment [5,6]. Since then, more and more scholars have applied social ecology theory to the study of the relationship between the living environment and the health of older adults [7,8].

Social ecology emphasizes the interaction between the environment, human behavior, and human health [5,9]. The effect of the community environment on the health of older adults is not isolated, but rather influences their behavior [10,11,12,13]. Previous studies have often used behavior as an intermediary variable for the impact of the community environment on the health of older adults. In these studies, the commonly used intermediary variable is the behavior of walking. More greenery, exercise facilities, and better leisure facilities will increase the walking frequency of older adults, which in turn will improve their health [10,11,12]. Meanwhile, the behavior of neighbor contacts has gradually been paid attention by scholars [14,15]. However, there are few studies on the comprehensive comparison of multiple behaviors in one model.

Self-rated health has been widely regarded as a dependent variable in previous studies of the direct impact of the community environment on the health of older adults [5,16,17,18,19]. However, there are relatively few studies on the effect of the living environment on the activity ability of older adults [20,21]. Moreover, there is a lack of comparative studies that incorporate both subjective (self-rated health) and objective (activity ability) indicators into the research framework. The activity ability refers to the basic and common activities that people carry out repeatedly every day in order to survive and adapt to the living environment. It is an important indicator of the health status of older adults [21]. In fact, activity ability is an effective proxy for older adults’ ability to move and live and has significant effects and implications on their life status and life quality [22,23]. Self-rated health is more likely to reflect older adults’ subjective feelings and satisfaction with their overall health [24,25,26]. The reflection of activity ability and self-rated health on the health of older adults has different emphases and values. Therefore, activity ability and self-rated health shed light on the different aspects of older adults’ health. However, it is not clear what relationships and differences exist between the effect of the community environment and behavior on the activity ability and self-rated health. As social ecology points out, the relationship between the environment, behavior, and human health occurs simultaneously, as do the effects of the community environment and behavior on the subjective and objective health of older adults. Therefore, the integration of activity ability and self-rated health into the social ecological model will help us to understand the relationship among the community environment and the behavior and health of older adults.

More importantly, due to the significant differences in the physiological function, behavior habits, and psychological state of older adults in different age stages, the effects of the community environment and behavior on their activity ability and self-rated health are also different. Studies that regard older people as a homogeneous whole tend to bias information about older people’s health impact factors [26,27], making it necessary to conduct comparative studies of older people in different age stages.

The research data of this paper is from the survey data of Shanghai, China. China is facing the typical rapid development of population ageing and urbanization. China’s big cities are also implementing a large number of practice projects, including urban renewal, old city renovation, and elderly oriented transformation of residential communities, which urgently needs the support of relevant theoretical research. Based on the theory of social ecology, this study used structural equation modeling (SEM) to analyze the relationships between the environment, behavior (including walking and neighbor contacts), and health of older adults from subjective health assessment and objective activity ability, and compared their similarities and differences. Meanwhile, we compared the relationship between the environment, behavior, and the health of older adults as a whole and between different age groups. We aimed to fully reveal the logic relationship among the community environment, walking, neighbor contacts, activity ability, and self-rated health of older adults. This will help scholars and even the majority of older adults to fully understand the real path of the community environment on individual health, and provide effective theoretical support for governments to build a truly age-friendly community.

## 2. Materials and Methods

### 2.1. Study Participants and Procedures

**Figure 1:**
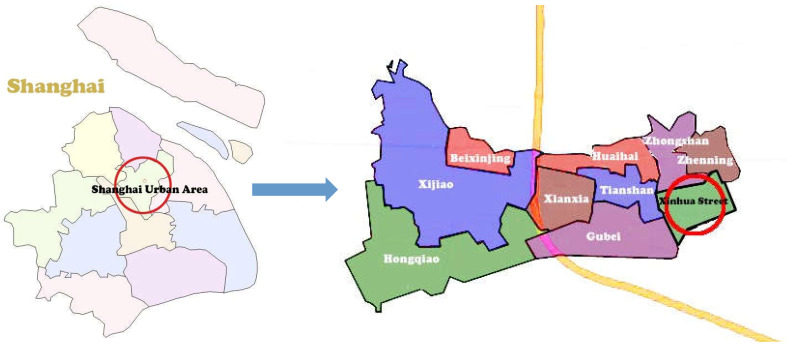
Location of Xinhua Street.

**Figure 2:**
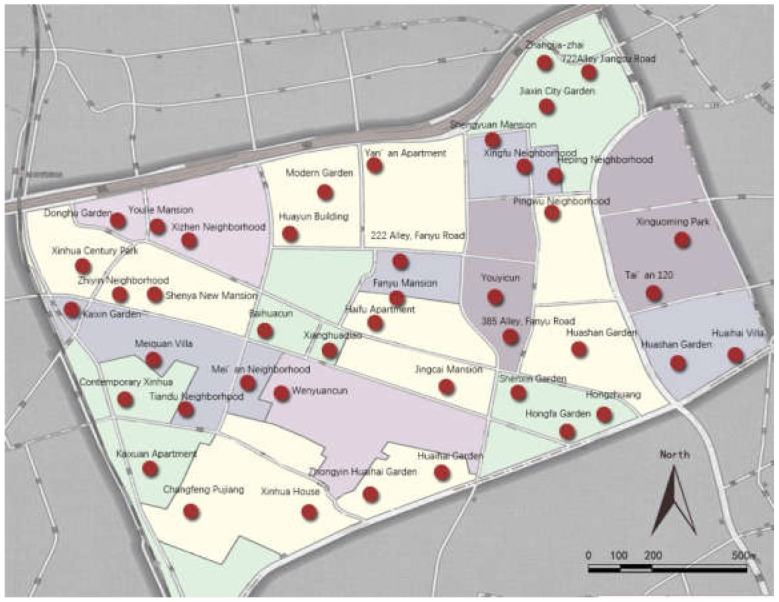
Map of the community sample.

The study was conducted among a sample of over-60-year-olds living in Xinhua Street, Changning District, Shanghai. The goal was to explore the relationship between elements of age-friendly communities, activities, and the well-being of older people. The survey was conducted in June 2014 and took six months. A two-stage sampling method was used in the survey. At the first step, we selected 43 out of the 198 residential areas in total in Xinhua street based on the diversity sampling principle in the location, quality, period, and scale, as shown in Figure 1 and Figure 2. At the second step, older adults aged 60 or above were sampled on a voluntary basis. For the residential areas with less than or equal to 120 samples, all of them were investigated. Otherwise, 120 samples were randomly selected to be investigated. From the total of 2839 samples, 2783 were validated. Of them, 1256 were men and 1627 were women, with an average age of 71.1 years. Based on their age, 1292 and 1491 were grouped as the younger elderly (60–69 years) and the older elderly (70 years and above), respectively, according to Matt and Dean (1993) [28] and Pinquart and Sorensen (2000) [29].

### 2.2. Variable Measurement

The measurement of the elderly’s self-rated health was obtained by asking respondents to rate their overall health with a score ranging from 1 to 5, which, from low to high, indicates poorer to better health.

Activity ability is often divided into two parts: activity of daily living (ADL) and instrumental activity of daily living (IADL). ADL focuses on whether older adults have functional disabilities, while IDAL tends to reflect the ability of older adults to live independently and adapt to social and family roles [23]. Since this research focuses on how the behavior and health of elderly people without functional disabilities are affected by the community environment, only IADL is used as a measure of activity ability of older adults [30,31].

There are seven items in the questionnaire for older adults’ activity ability, including shopping (0 = Not at all; 1 = need help from others to complete; 2 = can independently purchase daily necessities; 3 = can independently complete all shopping requirements), going out (0 = not at all; 1 = accompanied by others; 2 = can take a taxi independently; 3 = can take a bus independently; 4 = able to drive and cycle), cooking (0 = not at all; 1 = heat cooked meals by myself; 2 = make proper meals when materials are ready; 3 = can be done independently), housekeeping (0 = not at all; 1 = need assistance from others; 2 = unable to reach cleanliness; 3 = able to do simpler housework; 4 = can do more heavy housework), laundry (0 = not at all; 1 = can only wash small pieces of clothing; 2 = can wash all clothing independently), ability to use the phone (0 = not at all; 1 = only can answer the phone, not dialing; 2 = only the familiar phone can be dialed; 3 = can use the phone independently), and ability to handle finances (0 = not at all; 1 = need assistance from others; 2 = can handle finance independently). Due to the different judging criteria for different activity items, the grading of each item is not the same, as detailed in Table 1.

The community environment includes two parts: walking supportive environment and sensory supportive environment, which were measured using the two models developed by Mujahid et al. (2007) [32]. The walking supportive environment includes 7 aspects: suitable to walk, exercise opportunity provided, plenty of trees in the community, residents are attracted to walk, residents are attracted to exercise, exercise facilities provided, and convenient to walk. The sensory supportive environment includes three aspects: attractive environment, clean and neat community, and interesting buildings. All the aspects were graded from 1 to 5, and the description of variables and the sample situation are detailed in Table 1.

The behavior of older adults in this article was measured from two aspects of walking and neighbor contacts. Walking includes two variables: walking frequency and walking time. Neighbor contacts includes 5 items: help, communication, chat, care, and activity. The frequency of each item was indicated by 4 options, with a score of 1 to 4 representing “never, occasionally, sometimes, often”, respectively.

In this study, age, income, and education were included as control variables in the conceptual model. Among them, monthly income was indicated by one of the following six options: “<1500 CNY (Chinese Yuan), 1500–2500 CNY, 2500–3500 CNY, 3500–4500 CNY, 4500–5500 CNY, >5500 CNY”, scoring 1 to 6, respectively. Education is a 5-level item, with a score of 1 to 5 representing the following 5 options: “junior high school and below, senior high school, technical secondary school and technical school, junior college, undergraduate, master and above”, respectively.

### 2.3. Statistical Analysis

Our study adopted the statistical method of the structural equation model and used the maximum likelihood estimation method. The intermediary effect test is based on the MacKinnon, Lockwood and Williams (2004) [33] method.

Before formally analyzing the model path data, we tested the validity and reliability of the model variables and their fitness. The factor loadings of all observation variables reached the standard of 0.6. The composition reliability was greater than the standard of 0.6, and the average variance extraction was greater than the standard of 0.5 [34]. All the measured models had good reliability and validity suitable for SEM analysis. Because the correlation coefficient of the community sensory support environment and community walking support environment was 0.632, they together constituted the second-order model of the community environment.

GFI, AGFI, IFI, CFI, RMSER, and X^2^/DF were used to test the model fitness. The test criteria were GFI > 0.9, AGFI P > 0.9, IFI > 0.9, CFI P > 0.9, RMSER < 0.08, X^2^/DF < 5. The fitting result of the model showed that the fitness indexes GFI, AGFI, and RMSER meet the ideal standard, but IFI, CFI, and X^2^/DF did not, so the model needed to be optimized. After establishing the co-variation of the residuals e5 and e6, e9 and e10, e15 and e16, e16 and e17, and e28 and e29, all the indexes met the ideal standard.

## 3. Results

### 3.1. Descriptive Statistics of Samples

The descriptive statistics of the variables in Table 1 show that the activity ability and self-rated health of older adults gradually decreased with the increase of age. The mean values of all the observation variables in the community environment were similar to the mean values in different age groups. The duration and frequency of walking decreased with the increase of the degree of aging. However, for the older elderly group, the average duration of walking was 22.5 min and the frequency of walking was 3.44 times a week, suggesting a good walking habit relative to their age. The degree of neighbor contacts is generally low among the old people, but it is relatively lower for the older elderly group than for the younger elderly group. In the control variables, the income level and the education level showed a consistent law, that is, the younger elderly group was higher than that of the older elderly group.

### 3.2. Fitting Result of the Whole Sample Model

**Figure 3:**
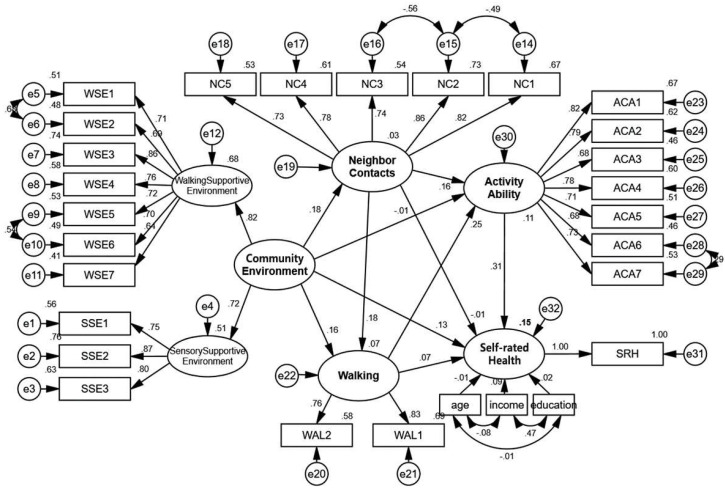
Standardized path diagram for the whole sample model.

When there are intermediate variables in the model, the relationship between independent variables and dependent variables should be interpreted more accurately in terms of the total effect, indirect effect, and direct effect. The total effect is equal to the direct effect plus the indirect effect. Among them, the total effect represents the total influence coefficient of the independent variable to the dependent variable. The direct effect represents the part that directly affects the dependent variable without passing through the intermediate variable. The indirect effect represents the coefficient of the independent variable’s influence on the dependent variable through the intermediate variable. In terms of the overall effect, older adults’ activity ability and self-rated health were positively and simultaneously influenced by the community environment, neighbor contacts, and walking. The overall effect values on activity ability were 0.069, 0.209, and 0.252, and those on the self-rated health were 0.159, 0.066, and 0.152, respectively (see Table 2 and Figure 3 for details).

The community environment has an insignificant direct effect and significant indirect effect on the activity ability of older adults. The mediating effect value is 0.029 and 0.048 for the neighbor contacts and walking, respectively. Both the direct and indirect effects of the community environment on the self-rated health of older adults were significant. The mediating effect values of walking and activity ability were 0.011 and 0.022, respectively.

### 3.3. Comparison of Model Differences between the Younger and Older Elderly Groups

**Figure 4:**
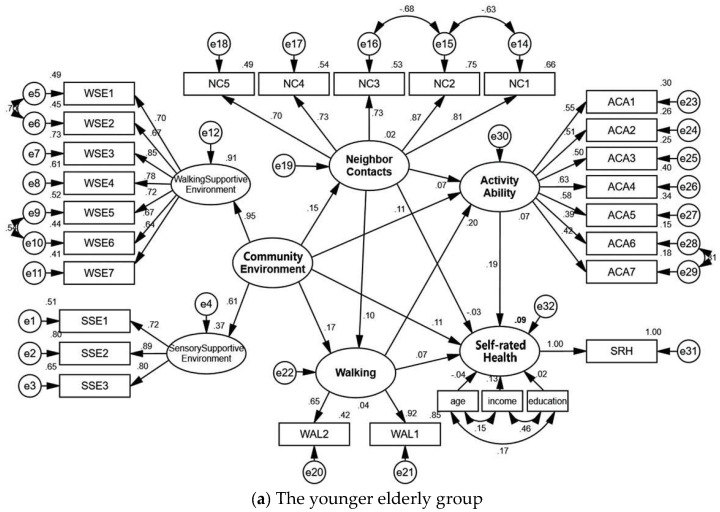

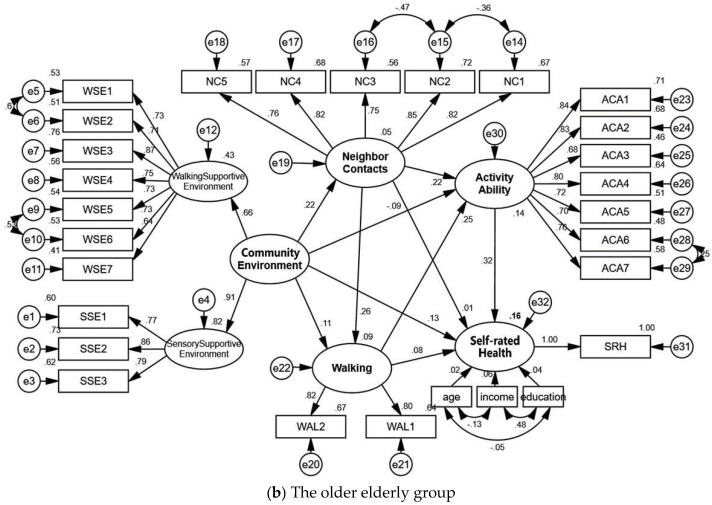
Comparison of models for different age groups.

The data of the younger group samples were introduced to the model to compare between age groups. The *p* value was <0.05 for the same path coefficient setting, suggesting there is a significant difference in the model path among different age groups. The standardized path map is shown in Figure 4 and the model paths are detailed in Table 3.

The activity ability of the younger elderly group was positively influenced by the community environment, neighbor contacts, and walking, with total effect values of 0.155, 0.091, and 0.201, respectively. The direct effect of the community environment and neighbor contacts on the activity ability of the younger elderly group was not significant, while the indirect effect was significant. The community environment, walking, and activity ability showed a significant positive effect on the self-rated health of the younger elderly group, with total effect values of 0.152, 0.107, and 0.192, respectively, while the neighbor contact did not. Both the direct and indirect effects of the community environment on the younger elderly group’s self-rated health were significant, but the direct effects of neighbor contacts and walking were not significant. Neighbor contacts and walking showed a significant positive effect on the activity ability of the older group of old people, with total effect values of 0.285 and 0.252, respectively. Community environment had no significant effect on their activity ability. The self-rated health of the older elderly group was significantly and positively influenced by the community environment, neighbor contacts, walking, and activity ability, with total effect values of 0.142, 0.126, 0.165, and 0.319, respectively.

## 4. Discussion

This study explored the relationship between the community environment, behavior, and the health of older adults. We focused more on the differences of this relationship at two levels (overall vs. age groups) and two dimensions (subjective health assessment and objective activity ability).

Our study confirmed the conclusion of some scholars that the community environment, walking, and neighbor contacts have significant positive effects on the activity ability and self-rated health of older adults [5,18,19,20,35]. In addition, our research found that the community environment and the behavior of older adults have different effects on their activity ability and self-rated health. These different effects are detailed as follows.

Firstly, the community environment has wider effects on older adults’ self-rated health, while behavior, including walking behavior and neighbor contacts, has a more intensive effect on the activity ability of older adults. This conclusion not only reveals the differences of the influence paths that the community environment and behavior affect the subjective and objective health of older adults in different dimensions, but more importantly, it provides a useful reference for us to improve and solve different targeted types of health problems of older adults. Secondly, the community environment has a more independent impact on older adults’ self-rated health. However, the influence of the community environment on the activity ability of older adults is realized completely through the mediating effect of behavior, including neighbor contacts and walking behavior. Therefore, in the design of a community environment, besides attention being paid to the community material environment, more attention should also be given to the design of the space and places that can effectively promote walking and interaction among older adults. In addition, guidance and publicity of healthy behaviors, such as walking and interaction, should be strengthened among older adults so as to more effectively contribute to the overall level of health of older adults.

Our study confirmed that there are differences in the impact factors and impact path of health of older adults in different age groups [27,36,37]. The community environment has a significant positive effect on the activity ability of the younger group of old people but not on that of the older group, which instead was significantly affected by the neighbor contacts. In other words, the health of the younger old people group is more easily influenced by the objective environment, while the health of the older group is more influenced by the social support provided by social interactions. The difference of the influence path of the self-rated health of older adults in different age groups is similar to that of the activity ability. Neighbor contact has a significant effect on the self-rated health of the older elderly group but not on that of the younger elderly group. The positive effect exerted by the activity ability on self-rated health is significantly less for the younger group than for the older group.

The difference of the model paths between older adults of the younger and older groups is of great theoretical significance for the related research on the health of old people, again strengthening the need for comparative studies of health determinants among different age groups of old people [26]. With the increase of global population aging as well as the life expectancy of older adults, the proportion of older adults is getting larger and larger. However, this huge group is not homogeneous as different age groups of older adults have huge differences in their psychological needs and behavior patterns. Therefore, to improve the overall health level of older adults, we need to put forward targeting advice and strategies based on different age characteristics. The younger elderly have a relatively good activity ability and health level, so the health problem of the older elderly often becomes the focus of research and improvement. According to our findings, to improve the health of the older elderly, particular attention should be paid to the support for neighbor contacts. The decision-makers and managers of the community environment can promote the neighbor contacts of the aged through various ways and means, such as providing support for social places, social information, and social services. This will not only help to improve the health level of the older elderly, but also help to realize the building of an age-friendly community, age-friendly city, and age-friendly society.

There are still some limitations in this study. First, as a cross-sectional study, we cannot fully explain the causative relationships between the community environment, neighbor interaction, and walking behavior, activity ability, and self-rated health of older adults. This needs the support of continuous longitudinal data from future studies. In addition, our examination of the community environment was based on subjective assessment. Therefore, our follow-up research will focus on exploring the relationship between the community environment and the health of older adults on the basis of the combination of subjective perception and objective measurement of the community environment, and comparing the differences and relationship between the subjective perception and the objective measurement of the community environment.

## 5. Conclusions

Our research introduced the social ecology theory into the study related to an age-friendly community environment, and compared the differences between the health paths of younger and older groups of old people. Our results clearly revealed the overall logical relationships between the community environment, behavior, and health of older adults. The effects of the community environment and behavior on the activity ability and self-rated health of older adults are different, and the path of the health influence of older adults is different in different age groups. Therefore, refined environmental governance and targeted improvement and resolution of different types of health problems among different groups of older persons will contribute to the overall health of older people and thus to the overall public health and well-being.

## Figures and Tables

**Table 1:** Characteristics of variables.

Latent Variables		Observed Variables	Mean (All)	Mean (The Younger Elderly)	Mean (The Older Elderly)
Dependent Variable Older Adults Health	Self-rated health (SRH)		2.35	2.52	2.08
	Activity Ability (ACA)	ACA1: Shopping	2.62	2.84	1.96
		ACA2: Going out	3.09	3.40	2.31
		ACA3: Cooking	2.53	2.76	1.90
		ACA4: Housekeeping	3.26	3.54	2.58
		ACA5: Laundry	1.67	1.84	1.23
		ACA6: Ability to use phone	2.87	2.97	2.48
		ACA7: Ability to handle finances	2.62	1.98	1.58
Independent Variable Community Environment	Walking Supportive Environment (WSE)	WSE1: Suitable to walk	3.31	3.33	3.32
		WSE2: Exercise opportunity provided	3.00	3.02	3.05
		WSE3: Plenty of trees in the community	3.14	3.11	3.25
		WSE4: Residents are attracted to walk	3.32	3.27	3.45
		WSE5: Residents are attracted to exercise	3.18	3.16	3.27
		WSE6: Exercise facilities provided	2.95	2.97	2.98
		WSE7: Convenient to walk	3.35	3.70	3.61
	Sensory Supportive Environment (SSE)	SSE1: Attractive environment	2.92	2.92	2.68
		SSE2: Clean and neat	3.61	3.61	3.65
		SSE3: Interesting buildings	2.72	2.72	2.76
Intermediary Variables Behavior	Neighbor Contacts (NC)	NC1: Help	2.37	2.45	2.13
		NC2: Communication	2.34	2.51	2.04
		NC3: Chat	2.44	2.51	2.17
		NC4: Care	1.91	1.96	1.77
		NC5: Activity	2.15	2.18	1.87
	Walking (WAL)	WAL1: Walking frequency	4.20	4.50	3.44
		WAL2: Walking time	28.6	31.25	22.5
Control variables		Income	3.33	3.30	3.22
		Education	2.24	2.17	1.95
		Age	72.45	67.19	83.57

**Table 2:** The model paths of the whole sample.

Independent Variable	Intermediary Variables				Dependent Variable					
	Neighbor Contacts	Walking			Activity Ability			Self-Rated Health		
		Total Effect	Direct Effect	Indirect Effect	Total Effect	Direct Effect	Indirect Effect	Total Effect	Direct Effect	Indirect Effect
Community Environment	0.182 ***	0.194 ***	0.160 ***	0.034 ***	0.069 **	−0.010	0.079 **	0.159 ***	0.126 ***	0.033 ***
Neighbor Contacts		0.184 ***	0.184 ***		0.209 ***	0.163 **	0.046 ***	0.066 **	−0.013	0.079 ***
Walking					0.252 ***	0.252 ***		0.152 ***	0.073 **	0.078 ***
Activity Ability								0.311 ***	0.311 ***	

Notes: *** means significant at the 0.01 confidence level; ** means significant at the 0.05 confidence level; the significance test chooses the percentile 95% confidence interval two-tailed test method.

**Table 3:** Comparison of the model paths in different age groups.

Independent Variable		Intermediary Variables				Dependent Variable					
		Neighbor Contacts	Walking			Activity Ability			Self-Rated Health		
			Total Effect	Direct Effect	Indirect Effect	Total Effect	Direct Effect	Indirect Effect	Total Effect	Direct Effect	Indirect Effect
The Younger Elderly	Community Environment	0.145 ***	0.183 ***	0.168 ***	0.015 ***	0.155 ***	0.108	0.047 ***	0.152 ***	0.114 ***	0.038 ***
	Neighbor Contacts		0.102 **	0.102 **		0.091 **	0.070	0.021 **	−0.007	−0.047	0.024
	Walking					0.201 ***	0.201 **		0.107 **	0.068	0.039 ***
	Activity Ability								0.192 ***	0.192 ***	
The Older Elderly	Community Environment	0.217 ***	0.164 **	0.107	0.057 ***	−0.005	−0.094	0.089	0.142 **	0.127 **	0.015
	Neighbor Contacts		0.261 ***	0.261 ***		0.285 ***	0.219 ***	0.066 ***	0.126 **	0.013	0.113 ***
	Walking					0.252 ***	0.252 ***		0.165 ***	0.085	0.080 ***
	Activity Ability								0.319 ***	0.319 ***	

Notes: *** means significant at the 0.01 confidence level; ** means significant at the 0.05 confidence level; the significance test chooses the percentile 95% confidence interval two-tailed test method.

## Data Availability

The data and samples in this study were collected by Fudan University in 2014. The ethical approval code number is IRB#2015-12-0574.

## Abbreviations

SEM	Structural Equation Modeling
CNY	Chinese Yuan
CFI	Comparative Fit Index
AGFI	Adjusted Goodness-of-Fit Index
IFI	Incremental Fit Index
GFI	Goodness-of-Fit Index
RMSEA	Root Mean Square Error of Approximation
X2/DF	Chi-Square/Degree of Freedom
ADL	Activity of Daily Living
IADL	Instrumental Activity of Daily Living
